# Construction of a dairy microbial genome catalog opens new perspectives for the metagenomic analysis of dairy fermented products

**DOI:** 10.1186/1471-2164-15-1101

**Published:** 2014-12-13

**Authors:** Mathieu Almeida, Agnès Hébert, Anne-Laure Abraham, Simon Rasmussen, Christophe Monnet, Nicolas Pons, Céline Delbès, Valentin Loux, Jean-Michel Batto, Pierre Leonard, Sean Kennedy, Stanislas Dusko Ehrlich, Mihai Pop, Marie-Christine Montel, Françoise Irlinger, Pierre Renault

**Affiliations:** Institut National de la Recherche Agronomique, UMR 1319 MICALIS, 78352 Jouy-en-Josas, France; AgroParisTech, UMR MICALIS, 78352 Jouy-en-Josas, France; Institut National de la Recherche Agronomique, US 1367 MGP, 78352 Jouy-en-Josas, France; AgroParisTech, UMR 782 GMPA, 78850 Thiverval-Grignon, France; Center for Biological Sequence Analysis, Technical University of Denmark, DK-2800 Kongens Lyngby, Denmark; Institut National de la Recherche Agronomique, UMR 782 GMPA, 78850 Thiverval-Grignon, France; Institut National de la Recherche Agronomique, UR 545 URF, 15000 Aurillac, France; Institut National de la Recherche Agronomique, UR 1077 MIG, 78352 Jouy-en-Josas, France; Department of Computer Science, Center for Bioinformatics and Computational Biology, University of Maryland, College Park, MD 20742 USA

**Keywords:** Genomic libraries, Genome sequencing, Sequence assembly, Next-generation sequencing, Comparative genomics, Metagenomics, Food bacteria, Dairy ecosystems

## Abstract

**Background:**

Microbial communities of traditional cheeses are complex and insufficiently characterized. The origin, safety and functional role in cheese making of these microbial communities are still not well understood. Metagenomic analysis of these communities by high throughput shotgun sequencing is a promising approach to characterize their genomic and functional profiles. Such analyses, however, critically depend on the availability of appropriate reference genome databases against which the sequencing reads can be aligned.

**Results:**

We built a reference genome catalog suitable for short read metagenomic analysis using a low-cost sequencing strategy. We selected 142 bacteria isolated from dairy products belonging to 137 different species and 67 genera, and succeeded to reconstruct the draft genome of 117 of them at a standard or high quality level, including isolates from the genera *Kluyvera*, *Luteococcus* and *Marinilactibacillus*, still missing from public database. To demonstrate the potential of this catalog, we analysed the microbial composition of the surface of two smear cheeses and one blue-veined cheese, and showed that a significant part of the microbiota of these traditional cheeses was composed of microorganisms newly sequenced in our study.

**Conclusions:**

Our study provides data, which combined with publicly available genome references, represents the most expansive catalog to date of cheese-associated bacteria. Using this extended dairy catalog, we revealed the presence in traditional cheese of dominant microorganisms not deliberately inoculated, mainly Gram-negative genera such as *Pseudoalteromonas haloplanktis* or *Psychrobacter immobilis,* that may contribute to the characteristics of cheese produced through traditional methods.

**Electronic supplementary material:**

The online version of this article (doi:10.1186/1471-2164-15-1101) contains supplementary material, which is available to authorized users.

## Background

Cheeses harbour a diverse microbial community, composed of a resident “house flora”, that interacts with strains deliberately inoculated as starter or adjunct cultures [1–5]. The cheese microorganisms mainly consist of Firmicutes (lactic acid bacteria, staphylococci), Actinobacteria (coryneform bacteria), Proteobacteria, Bacteroidetes, yeasts and moulds. Their concentration in the final product sometimes exceeds 10^10^ cells per gram and it is generally accepted that most of them are cultivable in laboratory growth media [6–9]. An inventory of microorganisms with a history of use in food fermentations was established recently [10]. It contains 195 bacterial species (30 genera) and 71 yeast and mould species (35 genera). Among these bacterial species, only 80 (41%) comprise at least one isolate for which a genome sequence isolated from food is available, with almost half of them within *Lactobacillus* species (NCBI database, May 2014). Furthermore, this list cannot be considered as exhaustive of the cheese microbial diversity, since occurrences of species previously undetected in milk and cheese are periodically reported [11–13] and isolates affiliated to novel taxa characterized [4]. Several species, such as *Corynebacterium casei*, *Microbacterium gubbeenense*, *Arthrobacter arilaitensis*, *Arthrobacter bergerei*, *Agrococcus casei*, *Mycetocola reblochoni* and *Vibrio casei* appear to be endemic in the cheese habitat and the environment of cheese manufacturing [14–17]. Environmental reservoirs of cheese microbial diversity such as milk, cow teat, human skin, brine baths, ripening room air, wooden vessels and shelves on which the cheese rests during ripening, have been identified, but their microorganism content remains largely uncharacterised [18, 19]. Microbial communities of cheeses and dairy environments also represent largely unexplored reservoirs of genetic and metabolic diversity with potential beneficial use for fermented food production. An increase in the number of genome sequences of dairy bacteria is also useful for a better understanding of the genetic determinants involved in the adaptation to the dairy habitat and the generation of functional properties [20–23].

In recent years, high-throughput sequencing technologies and information technologies have allowed the development of new approaches for studying the genetic diversity of microbial communities. Among these, metagenomics is a powerful tool for assessing the phylogenetic diversity of complex microbial assemblages present in samples such as soil, sediment, food products or water [24] and for exploring the functional properties of their dominant populations. The characterization of metagenomic datasets relies on the use of reference databases that contain sequences of known origin and phenotype. Many of these studies are carried out by pyrosequencing of single target genes, such as 16S rDNA sequences, that provide information restricted to the phylogenetic composition of the samples [25–31]. On the other hand, shotgun sequencing of whole community DNA provides additional information about the functions performed by the microbial community [32, 33]. The length of the reads generated by current high throughput sequencing technologies is too short to allow accurate comparative analyses against distantly related genomes, thus requiring the availability of reference genomes closely related to organisms from the environment being studied. Currently, the international genome databases are biased towards model organisms and pathogens, and, according to Huson et al. [34], up to 90% of the sequences of a metagenomic dataset may remain unidentified due to the lack of adequate reference sequences. The sequencing of several hundred genomes is no longer a technical issue. However, this process still remains costly, mainly due to the cost of the construction of individual libraries for each genome being sequenced.

In the present study, we selected and sequenced 142 bacterial strains of dairy origin that belong to 137 different species and 67 genera. In order to exemplify the relevance of these new genomes to the understanding of food microbiota, we used the newly created catalog to analyse the microbiota of three cheese surfaces sequenced through whole metagenomic sequencing.

## Results

### Creation of a dairy reference genome catalog

**Figure 1:**
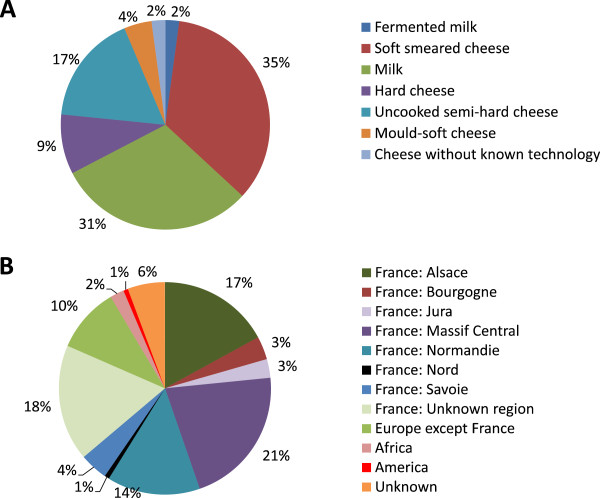
**Origin of the 142 selected dairy bacterial isolates in function of the type of dairy product (A) and the geographic area (B).**

After bibliographic investigation for bacterial species occurring in dairy products, we collected 142 dairy bacteria of various origins. The origin of the isolates and their taxonomy are shown in Additional file 1: Table S1. The collection comprised 36% Gram negative bacteria, 35% low GC Gram positive bacteria and 29% high GC Gram positive bacteria. Among the 67 corresponding genera, four are genera for which no genome sequence was available in NCBI databases (May 2014 release): *Kluyvera*, *Luteococcus*, *Marinilactibacillus*, and *Mycetocola*. The distribution of the strains according to the type of dairy product and their geographic origin is shown in Figure 1.

We designed a low-cost library sequencing strategy, by pooling the bacterial genomes together in a controlled way prior to sequencing in order to reduce library construction costs. Unlike other approaches that rely on barcoding, we de-convolve the individual genomes by using a co-abundance approach as described in Nielsen et al. [35] and Le Chatelier et al. [36] (see Additional file 2 supplementary document for an example of the clustering procedure). This strategy involves a two-step procedure, as follows. In the first step, the DNA of the 142 strains was mixed in five pools of about 30 strains each. To reduce the risk of chimeras each pool contained a mix of genomes from divergent genera (see Additional file 3: Table S2). The pools were sequenced using Illumina paired-end sequencing, and then assembled to produce five separate collections of contigs. In a second step, the genomes were redistributed into six pools of ~90 strains each, which were sequenced using SOLiD sequencing, and the resulting reads were mapped to the contigs generated in the first step in order to estimate the coverage of each contig within the stage 2 pools. We used SOLiD sequencing due to the availability of this platform at our institution and the lower cost of sequencing. However, other low-cost approaches for estimating the coverage of stage 1 contigs within the stage 2 pools could be used, such as, e.g., short runs on Illumina instruments. The resulting contig coverage matrix was then used to cluster together the contigs with correlated coverage profiles, each cluster corresponding to one of the original strains (see co-abundance clustering method section). The stage 1 pool assemblies contained each less than 20,000 contigs with a mean contig size of 40 kbp (see Additional file 3: Table S2, Illumina assembly pool sheet). After the clustering procedure, more than 80% of the Illumina contigs comprising more than 96% of the total length of contigs could be attributed to individual strains.

We assessed the quality of the clustering procedure by mapping the Illumina contigs to the closest NCBI genome sequences (using BLASTN [37], identity threshold > =90%). Furthermore, one organism in our collection - *Arthrobacter arilaitensis* Re117 – had already been sequenced and was added to one sequencing pool in order to validate the clustering procedure. We evaluated the correctness of contig clusters by computing two measures: the *dominant genus* - the percentage of the contigs that could be mapped to related genomes belonging to the same genus as the organism represented by the cluster; and the *reference coverage* - percentage of the total contig size of the pool that could be mapped to a genome from the dominant genus. We performed this analysis for the 53 draft genomes for which we could identify at least five genomes belonging to the same genus in the NCBI database. For these genomes, the mean dominant genus assignment and reference coverage percentage were of 97.7% and 89.5%, respectively (see Additional file 4: Table S3). The dominant genus assignment indicated that only 2.3% of the total length of the genomes may have been mis-assigned by the clustering procedure. The reference coverage indicated that on average 10.5% of the length of each genome may be missing. The potentially missing information is likely present in the 4% of the fragments that were not assigned and contain mostly repeated sequences. We performed an optimized re-assembly procedure for each draft genome, in order to increase contig size and recover eventual missing parts of the genomes (see Methods section). Interestingly, the optimized re-assembly process halved the number of contigs and increased slightly the total contig size of genomes for which close references were available, such as genomes of the genera *Leuconostoc* and *Streptococcus*. In order to further assess the quality of the draft genomes (including those without near-neighbors in public databases), we relied on the six quality submission criteria established by the Human Microbiome Project (HMP) [38], plus two additional criteria that identify potential miss-assignment events during the clustering step: phylogenetic marker redundancy and tetranucleotide homogeneity. For the HMP draft genome quality criteria, 5 criteria correspond to contig and scaffold assembly length and coverage (see Methods section). The last HMP quality criteria checks the presence of 99 bacterial essential genes [39], which gives an indication of the proportion of the genome that has been assembled (see Method section for the threshold used and supplementary information for the additional criteria). The phylogenetic marker redundancy tests the redundancy of 40 protein markers expected to be conserved in all bacteria, not laterally transferable and not duplicated within a genome [40]. The tetranucleotide homogeneity tests the homogeneity of the tetranucleotide signature among all the contigs of a draft genome. 101 genomes passed the essential genes HMP criterion and the two additional criteria for mis-assignment detection, indicating that 101 genomes are almost complete with no mis-assignment evidence (see Additional file 4: Table S3, draft quality evaluation sheet). Among these, 72 passed all the quality criteria and were defined as high quality draft genomes. An example is the genome of *Jeotgalicoccus psychrophilus* CRBM D2, which was assembled in only 70 contigs and 20 scaffolds, and had a contig N50 size of 103 kb. Sixteen additional assemblies presented incomplete sets of the HMP essential genes criterion but passed the two chimeric test criteria. For the 25 remaining draft genomes, 17 were too incomplete (<1 Mb in contig cumulative size) and 8 did not pass one of the two chimeric criteria. The genome of *Arthrobacter arilaitensis* Re117 [20], which was used in pool 1 as control for the procedure, passed all the HMP and chimeric criteria, and a comparison with the previously sequenced genome (Genbank project PRJNA53509) showed an average identity of 99.98%, a completion level of 95.63% and the absence of improperly assigned contigs. The two plasmids present in this bacterium were also partially present in our draft. The missing sequences corresponded mainly to transposase regions which were not assembled possibly due to the assembly and clustering procedure which often has difficulties reconstructing repeated variable regions. In total 117 of the draft genome sequences (101 which passed all quality controls and an Additional 16 that had no evidence of chimeric contigs) were considered suitable for submission to public databases (see Additional file 1: Table S1). The 25 draft genomes with poor quality or possible contamination were not submitted to public databases, but were used in our project with caution for phylogenetic analyses.

From the 195 bacterial species or subspecies listed by Bourdichon et al. [10] to occur in food products, only 80 had at least one food isolate for which a genome sequence was available in the NCBI database (NCBI May 2014 release, see Additional file 5: Table S4). The present study provides genome sequences for 78 additional dairy isolates, which effectively doubles the number of available genome sequences of relevance to the study of fermented dairy products.

**Figure 2:**
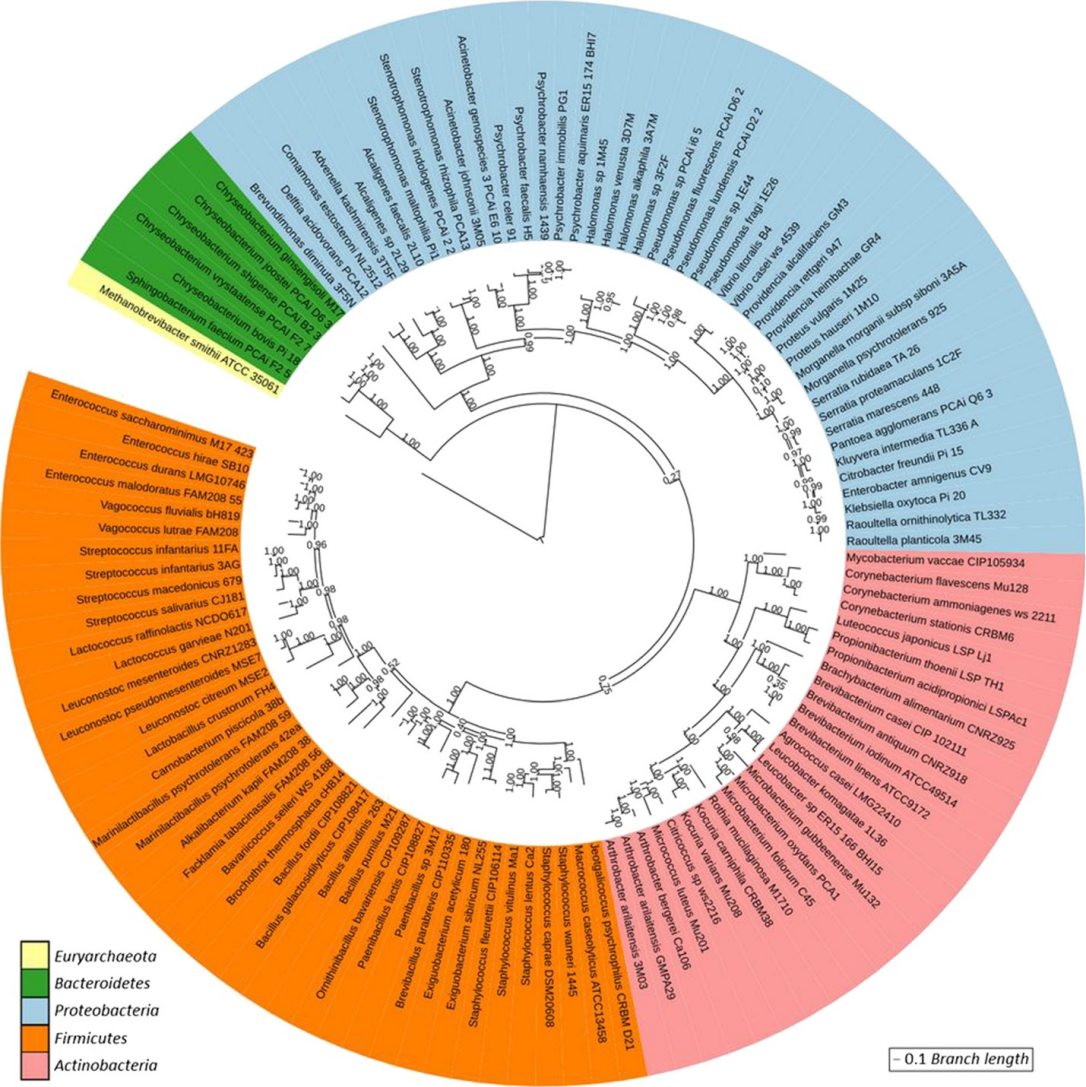
**Global phylogeny of the 117 dairy bacterial isolates sequenced in the present study.** The phylogenetic tree is an ITOL circular visualization [68] with the branch length and the bootstrap values displayed. The tree is based on a concatenated alignment of 40 universal marker protein families [40]. Only genome sequences from which a minimum of 10 markers could be extracted and which had no contaminating sequences evidence were considered. The genome of *Methanobrevibacter smithii* ATCC35061 was used to root the tree. The colors correspond to the different phyla.

In order to better characterize the diversity of the bacterial strains studied here, we reconstructed their phylogenetic relationships. For that purpose, the genes corresponding to the 40 phylogenetic protein markers proposed by Mende et al. [40] were extracted from the 117 high quality draft genomes in order to build a phylogenetic tree (Figure 2). The tree shows that the selected bacterial isolates cover a large biodiversity. Four other trees were constructed (see Additional file 6: Figure S1, Additional file 7: Figure S2, Additional file 8: Figure S3 and Additional file 9: Figure S4) by inclusion of 328 genomic sequences from food-related bacteria or closely-related species (Bacteroidetes, Firmicutes, Actinobacteria and Proteobacteria) extracted from the NCBI database (see Additional file 10: Table S5, genome references for phylogeny sheet). The classifications of the genomes sequenced in the present study are consistent with the NCBI reference genomes, further confirming the correctness of our reconstruction. In some cases, for example for *Alkalibacterium kapii*, *Marinilactibacillus psychrotolerans* and *Luteococcus japonicus*, only distant NCBI reference genomes are available, highlighting the contribution of our study.

### Genomics of cheese bacteria

The high quality draft sequences can be used to perform comparative genomic studies aimed at understanding the genetic underpinnings of the adaptation of bacteria to the food environment, e.g., through the characterization of metabolic pathways. Here we compared the genomes of strains from dairy and non-dairy environments for two genera. In a first example, we compared the genomic sequences of the four *Arthrobacter* strains isolated from cheese to that of 15 environmental isolates. Most bacteria of the genus *Arthrobacter* are isolated from environments such as soil, where they are considered to be ubiquitous [41]. Interestingly, the four cheese strains share several properties that may be linked to adaptation to the cheese habitat, such as a cluster of five genes involved in the catabolism of D-galactonate, as already described in *Arthrobacter arilaitensis* Re117 [20]. This gene cluster is absent from the genomes of the 15 *Arthrobacter* strains of environmental origin for which a sequence is available (see Additional file 11: Table S6). It has been hypothesized that D-galactonate may be produced by yeasts from lactose during the ripening of cheeses, and the ability to catabolize this compound could thus be beneficial for *Arthrobacter* strains in cheeses [20].

As a second example, we compared the genomes of two strains of *Streptococcus infantarius* isolated from Western African fermented milks, sequenced in this work (3AG and 11FA), with those of the type strain isolated from infant feces (ATCC BAA-102), and of strain CJ18, isolated from Eastern African fermented milk. The four strains contain each 1900–2000 genes and share 1567 genes. The strains could be divided into two groups, the two Western African strains, which displayed 99.7% identity on average within the shared genes (including 1206 fully identical genes), and the infant feces and the Eastern African strains, which displayed 99.3% identity (including 485 fully identical genes) (see Additional file 12: Table S7). The strains of the two different groups displayed only 98.8% identity and fewer than 185 fully identical genes, confirming a clear separation between the Western African food strain and the two others. Further study of the gene content of the two Western African *Streptococcus infantarius* strains showed that these strains had acquired the ability to ferment lactose through the LacZS system, as previously described for Eastern African strain CJ18 [42]. However, these genes are located within different regions of the chromosome and originate from a different donor. While *lacZS* may originate from *S. thermophilus* in the Eastern African CJ18 strain [43], it has probably been acquired from *S. salivarius* in the two Western Africa strains (see Additional file 13: Figure S5). These data show that adaptation of *S. infantarius* to the dairy fermentation niches occurred convergently and independently in these strains isolated respectively in Eastern and Western Africa.

### Application of the new genomic catalog to the metagenomic analysis of cheese microbiota

Metagenomic analyses based on sequence mapping on a set of reference genomes can be used to identify and quantify genes and species [36]. To determine whether the addition of the new genome sequences to the 5873 publicly available genomes (bacteria, archaea, yeasts and moulds) could improve such analyses, we sequenced the microbial communities from the rind of three cheeses with protected designation of origin. These three cheeses were made from cow’s milk and correspond to two soft smear-ripened cheeses (E and L), and one blue-veined cheese (G). Extracted DNA was sequenced by SOLiD technology and reads were assigned to species by mapping them to the reference microbial genomes and to the genome of *Bos taurus* (see Methods section).

**Figure 3:**
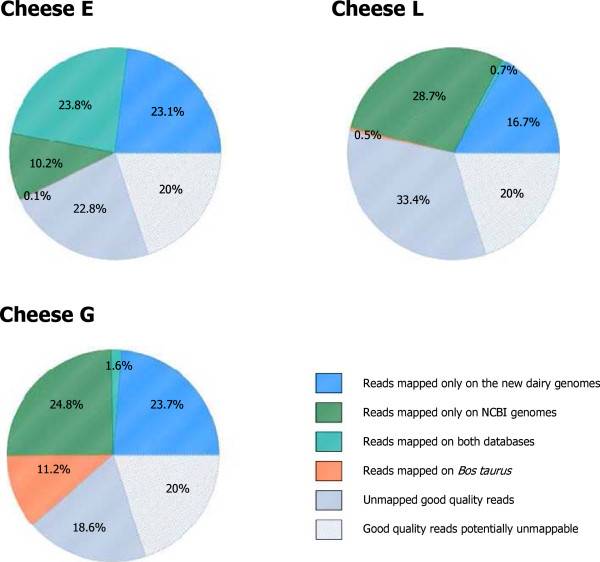
**Mapping of the good quality reads from the metagenomic sequencing of DNA from the surfaces of three cheeses.** The good quality reads coming from 3 samples of cheese surface were aligned to 5873 genomes coming from NCBI and 117 genomes coming from our project. The repartition of the good quality reads that map only on the NCBI genomes (blue), on the genome sequenced in our project (green), on both NCBI and our genome (light green) and on *Bos taurus* genome (orange) is presented in pie charts. The unmapped good reads are presented in dark and light grey, respectively those lacking a reference and those potentially unmappable for technical reason.

The sequencing of the three samples provided from 8.4 to 15.5 million good quality reads (see Additional file 14: Table S8). The percentage of good quality reads mapping to the microbial reference genomes varied from 46.1% (cheese L) to 57.1% (cheese E) (Figure 3). Interestingly, the reads that mapped only to the dairy genomes sequenced in the present study accounted for a large proportion of the good quality reads (from 16.7% for cheese L to 23.7% for cheese G). In cheese G, 11.2% of the reads mapped to the *Bos taurus* genome (compared to 0.5% and 0.1% for cheeses L and E, respectively). A deeper investigation of the cheese E good quality unmapped reads indicated that they may correspond to (i) strain specific genes belonging to the pan-genome (including prophages and mobile elements) and/or missing regions of the draft genomes, (ii) microorganisms for which genomes are still absent from the databases, (iii) distant genomic regions containing indels or more than 3 mismatches, which cannot be mapped with Bowtie. Lastly, ~20% of “technically good reads” on average are inherently un-mappable due to the characteristics of the SOLiD technology (this number is estimated from an analysis of the unmapped good quality read percentage in five different re-sequencing projects using the same sequencing and mapping techniques as in our paper, see Additional file 15: Table S9). The most prevalent microorganisms detected in the three cheeses are presented in Table 1 and a more detailed composition is shown in Additional file 14: Table S8.

**Table 1:** **Most prevalent microorganisms detected by metagenomic sequencing of three cheese surface samples**

	Reference genome	New(a)	Commercial cultures (b)	Number of reads (c)	Number of CDS (d)	Cumulated CDS length	Covered CDS (%) (e)	Covered sequence length (%) (f)	Mean genome coverage	Mapped reads (%) (g)	Sequences covered by perfect match reads (%) (h)
**Cheese E**	*Arthrobacter arilaitensis* GMPA29	**1**	**1**	2919327	3299	2875518	95.4	93.1	34.64	19.80	99.5
	*Psychrobacter immobilis* PG1	**1**	**0**	1259254	2941	2824830	99.4	94.3	15.48	8.54	99.9
	*Vibrio litoralis* B4	**1**	**0**	768119	3353	3158767	95.6	82.2	8.36	5.21	92.3
	*Pseudoalteromonas haloplanktis* TAC125	**0**	**0**	384444	3454	3327182	94.1	70.4	4.00	2.61	93.1
	*Geotrichum candidum* CLIB 918	**0**	**1**	378769	6925	10213537	98.3	38.6	1.30	2.57	95.5
	*Halomonas sp.* 1 M45	**1**	**0**	221008	3526	3281784	95.6	77.1	2.31	1.50	93.5
	*Lactococcus lactis* subsp. *lactis* Il1403	**0**	**1**	105179	2088	1869362	95.3	54.9	1.80	0.71	96.5
	*Debaryomyces hansenii* CBS767	**0**	**1**	77071	6295	9107395	91.7	16.6	0.30	1.27	85.6
**Cheese L**	*Pseudoalteromonas haloplanktis* TAC125	**0**	**0**	1434355	3454	3327182	93.7	89.5	14.95	17.03	97.6
	*Halomonas sp.* 1 M45	**1**	**0**	883652	3526	3281784	100.0	99.3	9.16	10.49	99.97
	*Psychrobacter celer* 91	**1**	**0**	146974	2584	2414775	95.6	76.5	2.08	1.74	91.5
	*Lactococcus lactis* subsp*. cremoris* A76	**0**	**1**	143400	2470	1969568	92.5	35.4	0.82	1.70	98.1
	*Penicillium camemberti* FM 013	**0**	**1**	83277	14611	22234696	85.1	10.8	0.13	0.99	97.7
	*Vibrio litoralis* B4	**1**	**0**	65937	3353	3158767	90.0	27.6	0.70	0.78	85.9
	*Providencia heimbachae* GR4	**1**	**0**	53280	3824	3516737	83.6	26.7	0.51	0.63	55.3
	*Geotrichum candidum* CLIB 918	**0**	**1**	45165	6925	10213537	95.0	12.7	0.15	0.54	95
**Cheese G**	*Arthrobacter bergerei* Ca106	**1**	**1**	2878361	3553	3110335	98.6	95.7	31.77	18.58	99.2
	*Lactobacillus delbrueckii* subsp*. bulgaricus* ATCC11842	**0**	**1**	1179878	1508	1340406	98.7	95.4	29.44	7.62	99.8
	*Penicillium camemberti* FM 013	**0**	**1**	633123	14611	22234696	99.4	54.2	1.00	4.09	96.5
	*Streptococcus thermophilus* LMG 18311	**0**	**1**	597916	1827	1462709	98.3	86.1	13.62	3.86	99.4
	*Penicillium roqueforti* FM 164	**0**	**1**	254435	12630	23447373	98.5	26.3	0.38	1.64	98.4
	*Pseudoalteromonas haloplanktis* TAC125	**0**	**0**	146457	3454	3327182	93.5	57.8	1.53	0.95	92.3
	*Debaryomyces hansenii* CBS767	**0**	**1**	80179	6295	9107395	94.2	17.6	0.31	0.52	97.1
	*Psychrobacter aquimaris* ER15 174 BHI7	**1**	**0**	74153	2830	2734881	93.7	41.7	0.87	0.48	81.5

(a) Genomes sequenced in the present study (1) or from the NCBI database (0).

(b) Species known to be components of cheese making commercial cultures.

(c) Number of reads mapped on CDS from the reference genome with three or less mismatches on 35 nucleotides.

(d) Number of CDS in the genome. CDS corresponding to insertion sequences, prophages and potential repeated and transferable elements were removed.

(e) Percentage of CDS covered with at least one read.

(f) Percentage of sequence covered by at least one read (sequence is restrained to the selected CDS).

(g) Number of reads aligned with this genome divided by the number of good quality reads.

(h) Length of sequence covered by perfect match reads (with no mismatch on the 35 nt length alignment) divided by the length of the sequence covered by reads.

For each cheese, the data presented correspond to the eight reference genomes with the highest numbers of mapped reads.

In the smear-ripened cheese E, the *Arthrobacter arilaitensis* GMPA29 reference genome was the most represented, as it corresponded to 19.8% of the total good quality reads, followed by *Psychrobacter immobilis* PG1 and *Vibrio litoralis* B4, with 8.5 and 5.2% of the reads, respectively. Furthermore, between 95.4 and 99.4% of their coding sequences were detected, with a high level of coverage (from 8.4X to 34.6X). The genomes of *Arthrobacter arilaitensis* GMPA29 and *Psychrobacter immobilis* PG1 are almost entirely covered (from 99.5 to 99.9% of the reference size), showing that the detected strains were closely related to the reference strains. The reference strain *Psychrobacter immobilis* PG1 was isolated from the dairy plant that produces the smear-ripened cheese E, but two years earlier. The high proportion of perfect matches with reference strain PG1 may thus be explained by the presence of an offspring of this strain in cheese E. Many reads were also assigned to the genomes of the yeasts *Geotrichum candidum* CLIB 918 and *Debaryomyces hansenii* CBS767.

In the second smear-ripened cheese (cheese L), *Pseudoalteromonas haloplanktis* TAC125, *Halomonas* sp. 1 M45 and *Psychrobacter celer* 91 were the three dominant reference bacteria, with 17.0%, 10.5% and 1.7% of the good quality matches, respectively. In this cheese sample, the sequences of the reads mapped to *Halomonas* sp. 1 M45 were essentially perfect matches (>99.9% of covered positions with 99.9% perfect match reads, coverage of 9.2X). These data suggest that the *Halomonas* strains present in the cheese sample are almost identical to the reference strain. However, even though the reference strain 1 M45 has also been isolated from a smear-ripened cheese of the same protected designation of origin, it originated from another manufacturing plant. More than fifty thousand reads were assigned to *Providencia heimbachae* GR4. However, only 55.3% of covered positions of this reference strain are without mismatch, which indicates that the strain present in the cheese sample is not closely related to the reference strain, and may even correspond to another species. Surprisingly, more than 80 thousand reads (~1% of the total good quality reads in cheese L) mapped to the *Penicillium camemberti* FM 013 genome, with 97.7% of perfect match reads, even though this species is not known to occur in smear-ripened cheeses. One may hypothesize that this could result from cross-contamination due to the manufacturing of mould-ripened cheese in the same plant.

The surface of the blue-veined cheese G was dominated by a strain close to *Arthrobacter bergerei* Ca106 (18.6% of the reads, 99.2% perfect match reads). Like for the two other cheeses, *Psychrobacter* species seem to be present in this cheese. Cheese G was probably manufactured with a thermophilic lactic starter culture, since *Streptococcus thermophilus* and *Lactobacillus delbrueckii* species were the dominant lactic acid bacteria, in contrast to the two other cheeses, in which *Lactococcus lactis* was the dominant lactic acid bacterium. Strains related to other reference strains sequenced in the present study, such as *Psychrobacter aquimaris*, *Brachybacterium tyrofermentans*, *Corynebacterium ammoniagenes*, *Brevibacterium antiquum*, *Microbacterium gubbeenense*, *Brochothrix thermosphacta* and *Marinilactibacillus psychrotolerans*, were also present in the cheeses (>80% perfect match reads, see Additional file 14: Table S8)*.* Interestingly, among the eight most prevalent microorganisms detected in each cheese by metagenomic analysis, two (cheese G), four (cheese E) or five (cheese L) corresponded to species or genera of gram-negative bacteria which are not known components of cheesemaking commercial cultures (Table 1).

## Discussion

In the present work, we produced 137 draft genomes isolated from dairy products, which almost doubled the number of different species isolated from fermented dairy products. This genome catalog was realized using a low-cost library sequencing strategy based on a combinatorial pooling approach in which a reduced number of DNA pools were sequenced. Pooling strategies have been previously been used to reduce costs for BAC sequencing [44]. Here we rely on a co-abundance clustering approach that we developed for reconstructing genomes directly from metagenomic samples [35]. In the present pooled approach, only 11 libraries were required to produce 150 draft genomes, leading to a cost of ~200 USD per genome as opposed to ~500 USD if each genome were sequenced separately (using best commercial offers available in 2011). Today, this price differential may be even higher as library construction costs have not decreased as much as sequencing costs. This cost savings comes with some limitations. First, we suggest that only distant genomes (*i.e.* from different genera at least) should be mixed and sequenced together to optimize the assembly and clustering steps. Second, several genomes were poorly sequenced, however most of them were high GC% draft genome, known to be difficult to sequence using Illumina sequencing [45]. Also, about 4% of the total sequence length could not be assigned to an individual strain and we estimated that on average 2% of a genome’s sequence may be mis-assigned due to limitations of the clustering approach. However, an examination of unassigned fragments > 2 kb showed that they correspond mainly to mobile elements (plasmids and phages) while genome data curation showed that potentially mis-assigned fragments are generally < 1 kb, and primarily impact genomes of lower quality. This fact prompts us to suggest that the use of these drafts for comparative genomics should be restricted to high quality draft genomes and to genes present in long contigs or scaffolds (*i.e.* bigger than 1 kb). Lastly, the bioinformatics analysis pipeline is more complex to set up than simple assembly procedure in single genome sequencing. Despite these limitations, 117 of the 142 sequenced genomes resulted in good or high quality draft genomes suitable for submission to public database. Some of the remaining 25 genomes may still be useful as references for metagenomic analyses.

The microbial composition of the surfaces of three cheeses was investigated by high throughput metagenomic profiling. As all the DNA present in the cheese samples is sequenced, the high throughput sequencing may detect any type of DNA (bacteria, archaea, eukaryotes and viruses), provided that adequate references are used. Eukaryotes, such as *Geotrichum candidum*, *Debaryomyces hansenii*, and *Penicillium roqueforti*, were found, which was not surprising, as these fungi are frequently used in cheesemaking. Interestingly, many reads from cheese G mapped on the *Bos taurus* genome. We hypothesize that this is due to the presence of cow somatic cells in the milk used for the manufacturing of cheese G. The impact of milk somatic cells on the ripening of cheeses has been shown in several studies as associated to the flock health [46, 47].

Shotgun sequencing allows a relative quantification of DNA molecules present in a sample, based on counting the number of reads mapped to each member of the community. High throughput sequencing allows higher resolution quantitation and we have shown that we can recover even fairly minor taxonomi groups, such as the *Leuconostoc* genus (see Additional file 14: Table S8), known to be part of the minority population in cheeses [48]. However, additional experiments may be needed to validate the identification and quantitation of low abundance populations.

Presence or absence of complete set of genes or of specific genes, and their level of sequence homology allows also confirming characteristics of particular strains. For example, cheeses E and L metagenomic profiles indicated the presence of strains closely related, respectively, to *Psychrobacter immobilis* PG1 and *Halomonas* sp. 1 M45 coming from our catalog. Since these reference strains were isolated in the same cheese factory several years earlier for the former and in the same DOP from another factory for the later, our analysis would reflect the setting up of strains sharing common origins with the references in these cheeses. Metagenomic profiling provides thus new perspectives to study cheese ecology by tracing genomes or genes, which should allow pointing out particular strains (*e.g.* starters, potential “terroir” or regional strains, contaminants…), and following their dissemination and development during cheese processes.

The metagenomic profiling of the surfaces of the three cheeses confirmed that microorganisms that are not deliberately inoculated constitute a large part of the microbiota, appearing among the few dominant species in cheese rind. For example, they are predominant in cheese L. Several of the corresponding microorganisms, such as *Pseudoalteromonas*, *Halomonas, Vibrio, Marinilactibacillus* and *Psychrobacter* are Gram-negative bacteria which had been previously detected in such cheeses[1, 2, 9, 12, 13, 28, 49–53], and also in a recent large amplicon sequencing study of the microbial composition of 137 different cheese rinds [33]. They may originate from the environment of cheese manufacturing (brine, tools, surfaces of shelves …), and their high abundance suggests that they may have an impact on the properties of the final product. As the analysed cheeses were marketed and were of very good quality, these microorganisms cannot be considered here as spoilers.

## Conclusion

The genomes sequenced in the present study considerably increased the numbers of mapped reads, although a significant proportion of the metagenomic reads remained unassigned (~20% once taken into account unmapped reads inherent to SOLiD technology). These data indicate that even if more than 6000 genomes are currently available in public databases (including the genomes we generated here), additional microorganisms found in traditional cheeses are still missing from this reference collection. Further studies are necessary to complete this reference in order to provide a complete view of the cheese ecosystem. To our surprise, collecting the present reference strain set constituted a laborious work, since strains corresponding to non-starter species are frequently not conserved once described. We anticipate that the results of this work will motivate isolation and conservation of new reference strains, as well as independent isolates of the same species to support safety assessment, establish biodiversity resource and strain specificity in products. Direct sequencing and assembly as performed for the human microbiome could also provide new potential references [36], although this procedure is significantly more expensive. In summary, the present study considerably extended the effectiveness of shotgun metagenomic analysis of cheese microbiota. Even if such analyses require generating and computing large amounts of sequencing data, the technologies are evolving rapidly and one may anticipate that in the future, they will become routine in the investigation of food microbiota.

## Methods

### Bacterial isolates and growth conditions

The bacterial isolates working collection was composed of 142 isolates originating from milk, fermented milks, and cheeses, and five food isolates that were not of dairy origin (see Additional file 1: Table S1).

### DNA extraction from liquid cultures of bacterial isolates

After cultivation, bacterial cells were harvested by centrifugation for 10 min at 12,000 × *g* and approximately 100 mg of cell pellet were suspended in 400 μl of buffer (0.4 M NaCl, 2 mM EDTA, 10 mM Tris–HCl, pH 8). For gram-positive bacteria, an enzymatic lysis step was performed by incubating the cells for 1 hour at 37°C after addition of 50 μl of lysozyme (20 mg/ml) for *Actinobacteria* strains or of 50 μl of lysostaphin (100 Units) for *Staphylococcus* strains. One hundred microliters of SDS (20%) and 40 μl of proteinase K (15 mg/ml) were then added and the mixture was subsequently incubated for 1 hour at 55°C. One hundred and fifty mg of 0.1 mm-diameter zirconium beads (Biospec Products, Bartlesville, OK, USA), 150 mg of 0.5 mm-diameter beads and 500 μl of phenol/chloroform/isoamylic alcohol (25/24/1; pH 8) were added to the tube, which was vigorously shaken in a bead-beater (FastPrep-24 instrument; MP Biomedicals Europe, Illkirch, France) for 45 s at a speed of 6.0 m/s. The sample was centrifuged (45 min at 12,000 × g) and the upper phase was transferred in a Phase Lock Gel-heavy tube (Eppendorf, Hamburg, Germany) and mixed with 500 μl of phenol/chloroform/isoamylic alcohol. After centrifugation (15 min at 12,000 × *g*), the upper phase was mixed with 500 μl of chloroform and centrifuged (15 min at 12,000 × *g*). DNA was precipitated overnight at −20°C after addition of 0.1 volume of 3 M sodium acetate and 2 volumes of cold absolute ethanol to the upper phase. After centrifugation (30 min at 12,000 × *g*), the DNA pellet was washed with 70% ethanol and resuspended in 1X TE buffer. Two microliters of RNase solution (10 mg/ml) were added and the mixture was subsequently incubated for 30 min at 37°C. The concentration and quality of genomic DNA was evaluated using a NanoDrop ND-1000 spectrophotometer (NanoDrop Technology Inc., Wilmington, DE, USA). Moreover DNA (5 μL) was loaded on a 1% agarose gel and visualized after migration by ethidium bromide staining.

### Sequencing of rrs and rpoB genes from bacterial isolates

The species corresponding to each genome was confirmed by sequencing the 16S rRNA or the *rpoB* gene (see Additional file 1: Table S1). The *rrs* gene (encoding the 16S rRNA) was amplified with primers pA (5′-AGAGTTTGATCCTGGCTCAG-3′) and pH (5′-AAGGAGGTGATCCAGCCGCA-3′), as previously described [54]. Since the rrs gene is not always sufficient to distinguish closely related species, especially *Enterobacteriaceae*, the housekeeping gene *rpoB* (encoding the beta chain of the DNA-directed RNA polymerase), that has been shown to resolve phylogenetic relationships in various bacterial groups [55], was also used. PCR amplification of *rpoB* was performed with primers VIC4 (5′-GGCGAAATGGCDGARAACCA-3′) and VIC6 (5′-GARTCYTCGAAGTGGTAACC-3′) [56]. Both strands of the resulting amplicons were sequenced by GATC Biotech (Konstanz, Germany), using the same primers than for the PCR amplifications. The sequences were then assembled using the CAP3 program [57] and compared to the GenBank database using the Basic Local Alignment Search Tool (BLAST) [37] to determine the closest known relatives of the *rrs* or *rpoB* gene sequences.

### Genome sequencing

The 147 bacteria DNA samples were distributed in equivalent amounts (0.3 μg) in five metagenomic pools containing each about 30 genomes. Only different genera were mixed together in a single pool to improve the assembly process by reducing the presence of possible identical regions (see the Additional file 2 supplementary information document). Each pool was sequenced using the Illumina HiSeq 2000 system, with around 90 million paired-end reads of 91 nucleotides in length, an insert size of ~350 bp for pool 1 and ~310 bp for the four other pools. Low quality reads (with 3 or more “N”), which constituted less than 8% of the total reads, were discarded. The assembly was performed for the five metagenomic pools independently, using SOAPdenovo (v1.04) [58]. Kmer size was selected separately for each pool by evaluating the best contig size, N50 and N90, and the best percentage of reads re-mapped to the assembly (see Additional file 3: Table S2, Illumina assembly sheet). Only contigs of 100 bp in length or more were kept for further analysis. The draft genomes are available under the pending BioProject ID PRJEB230 to PRJEB363 (see Additional file 1: Table S1).

### Co-variance clustering

To assign the contigs to their original genomes, we used a clustering method based on the co-variance principle, derived from method described by Nielsen et al. [35] and Le Chatelier et al. [59] (see the Additional file 2, supplementary information). For this purpose, six new DNA pools were created by mixing genomic DNA so that each DNA sample has a different combination in the pools, and therefore each genome has a unique presence signature (see Additional file 3: Table S2, composition pool sheet). The six new DNA pools were sequenced using SOLiD technology 4, with around 90 million single reads in each pool of 50 nucleotide length. The SOLiD reads were aligned to the five Illumina contigs using the Bowtie aligner [60] (see Additional file 3: Table S2, SOLiD mapping data sheet). This resulted in the creation of five independent contig coverage matrix, used for the clustering process. This alignment allows calculating a coverage vector for each contig, corresponding to the presence and absence of the contig in the 6 combinatorial pools. The contig coverage vectors are compared to the strain presence and absence in the 6 combinatorial pools, using the Pearson correlation coefficient. The contigs are assigned to the strain with which they share the highest Pearson correlation, as long as the correlation value is equal to or higher than 0.95. The Illumina and SOLiD samples are accessible under the project ID PRJEB6314 at the SRA database.

### Genes and contigs taxonomical annotation

All contigs were taxonomically annotated by sequence similarity using BLASTN to a database containing 1411 reference genomes (extracted from NCBI database, June 2012 version). Sequence similarity > =90% on at least 100 nucleotide was used for genus level annotation. This taxonomical assignment was used to calculate the dominant genus assignment and the reference coverage of the clustering, by considering the 53 strains with at least 5 different species present in the NCBI database. This restriction was made in order to reduce the likelihood of incorrect assignments by the BLASTN procedure (see Additional file 4: Table S3, cluster BLASTN assignation sheet). The unassigned contigs were considered as part of the dominant genus assignment, to differentiate them from the contigs assigned to a different genus which represent a potential mis-assignment from the clustering step. The 11 clusters with less than 500 kb in total contig size were not considered for the dominant genus assignment and reference coverage due to their low quality and high level of fragmentation.

### Optimize re-assembly of draft genomes and quality evaluation

To increase the size of contigs and scaffolds, we performed an optimized de novo re-assembly procedure after the clustering procedure. The contig pools were used to recruit reads from their Illumina HiSeq 2000 sequencing pool, by alignment to the contigs using BWA [61]. The recruited reads were corrected using Quake [62]. A new de novo assembly was performed for each cluster using only the reads that were remapped on them, using Velvet [63]. Scaffold gaps were filled using SOAPdenovo GAPCloser [58]. When a close species reference genome was available in the NCBI database, we used it for an assisted assembly procedure. This procedure used the NCBI contigs in combination with the cluster contigs to recruit the reads. This may recover the missing regions that were lost during the first assembly and clustering procedures. Reconstructed genome drafts were classified from low to high quality by using the Human Microbiome Project assembly criteria [64]. The threshold used for each HMP criteria validation are: (1) contig N90 > = 500 bp; (2) 90% or more of the 99 bacterial essential genes are found; (3) 90% or more of the bases in the assembly have more than 5 fold sequence coverage; (4) contig N50 > = 5 kb; (5) scaffold N50 > = 20 kb; (6) average contig length > = 5 kb. Two other additional criteria for chimera detection were computed: the tetranucleotide homogeneity score and function redundancy (see Additional file 4: Table S3, draft genome quality sheet; and the supplementary information document). The tetranucleotide homogeneity was calculated by counting the number of different tetranucleotides in all contigs, divided by the size of the contigs, to produce a tetranucleotide frequency vector. Each tetranucleotide frequency vectors were compared using the Spearman rho correlation, and the mean rho correlation value of all pairwise Spearman correlation comparison was calculated. The draft were considered as potentially contaminated by another draft when the mean Spearman rho was lower than 0.6. The 40 marker protein redundancy was calculated by first searching for the marker protein in the draft as described in the Genome annotation and phylogeny annotation procedure section. Then each protein detected at least twice was listed in all drafts. A draft was considered as chimeric if 3 or more markers were redundant.

### Genome annotation and phylogeny annotation procedure

RAST (Rapid Annotation using Subsystem Technology) was used for annotating the genome drafts in the SEED environment [65]. Genomic drafts passing the core-ratio HMP criterion and 328 NCBI reference genomes were used for phylogenic classification (see Additional file 10: Table S5, genome references phylogeny sheet). For each draft, 40 markers commonly used for phylogeny classification and corresponding to 40 essential proteins [40] *,* were detected using BLASTP procedure and a marker reference database of about 1500 complete genomes. The best hit was selected with at least 50% identity and 50% coverage. Each marker protein was aligned to reference markers using MUSCLE [66] and the 40 individual alignments were concatenated to a single alignment. The missing markers were replaced by gapped lines, not used for the distance calculation. Only the drafts with at least 10 markers out of the 40 described above were selected for the tree. The tree was constructed using FastTree [67] with the parameters: −*gamma -pseudo -spr 4 -mlacc 3 -slownni*. The visualization was done using ITOL [68], in circular mode view and branch length displayed mode.

### Extraction of DNA from cheese samples

For each of the three types of cheeses, five pieces of rind (8 cm^2^; mean thickness: ~5 mm) were taken using a circular punch (3.2 cm in diameter) and a knife, mixed together, and cut in small pieces. Twenty grams of cheese rind were then mixed with 20 ml of guanidium thiocyanate (4 M) in Tris–HCl (pH 7.5, 0.1 M) and dispersed with a mechanical blender (Ultra-Turrax® model T25; Ika Labortechnik, Staufen, Germany) for 3 min at 14,000 rpm. After adding 2.4 ml of sodium laurylsarcosinate (100 g/l) and gentle mixing for 1 min, five 1.9 ml-aliquots were added to five 2-ml tubes containing 350 mg of zirconium beads (0.1-mm diameter; Sigma, St-Quentin-Fallavier, France) and the tubes were centrifuged for 10 min at 20,800 *g* and 4°C. The supernatants, which included the fat layer, were removed, and the tubes were frozen at −20°C. After thawing, 600 μl of Tris-EDTA buffer (pH 8.0; 100 mM Tris, 10 mM EDTA), 40 μL of proteinase K (15 mg/ml; Sigma, St Quentin Fallavier, France) and 100 μl of sodium dodecyl sulfate (200 g/l) were added to the tubes, which were then vortexed for 2 min, and subsequently incubated for 2 h at 55°C. After cooling on ice, the tubes were vigorously shaken for 40 s in a bead beater (FastPrep®-2 System; MP Biomedicals, Illkirch, France) at a speed of 4.0 m/s. 700 μl of phenol were then added and the content of the tubes was gently mixed for 1 min. The tubes were centrifuged for 5 min at 20,800 *g* and 20°C and the aqueous phases were transferred to 2-ml tubes containing a gel that improves separation between the aqueous and organic phases (Phase Lock Gel™ Heavy; Eppendorf, Germany). After adding 700 μl of phenol-chloroform-isoamyl alcohol (25:24:1; saturated with 10 mM Tris, pH 8.0, 1 mM EDTA) and gentle mixing for 1 min, another centrifugation was performed for 5 min at 20,800 *g* and 20°C and the aqueous phases were transferred in new Phase Lock Gel™ tubes. Another extraction with 700 μl of phenol-chloroform-isoamyl alcohol was then performed, and the aqueous phases were recovered in a 2-ml centrifugation tube, mixed with 5 μl of RNase A (20 mg/ml, Sigma), and incubated for 1 h at 37°C. The DNA was then precipitated by adding 1 ml of isopropanol and 50 μl of sodium chloride (5 M) and incubating the tubes overnight at −20°C. The DNA was recovered by centrifugation for 10 min at 20,800 *g* and 4°C, and the pellets were subsequently washed three times with 1 ml of 70% (vol/vol) ethanol. They were then dried for 30 min at 42°C and dissolved in 50 μl of water, after which the five samples corresponding to the same cheese were pooled together.

### Metagenomic sample mapping

Three DNA samples from cheeses were sequenced using SOLiD technology, which yielded between 11 and 19 million single reads of 50 nucleotides length. The identification of species was done in two steps: a first mapping was done on a catalog reference of 5990 genomes, which included the 117 draft sequenced in the present study, and a second mapping with more detailed analyses on a selection of genomes (see Additional file 10: Table S5, genomes metagenomic analyse sheet). The first mapping was performed using Bowtie aligner [60] (with parameters: first 35 nucleotides mapped; 3 mismatches allowed; 10000 matches by read allowed), on the whole database. This mapping allowed a first selection of genomes. Genomes with no annotation were cut into fragments of 1000 bases. Genomes with less than 20% of genes (or less than 20% of fragments) covered by reads were removed. For species expected to be in food microbiota, several reference genomes were chosen. Otherwise, one genome was selected for each species. When possible, genomes with annotations were selected in priority. The samples were then mapped against the selected genomes (59 for cheese E, 86 for cheese L, 67 for cheese G; see Additional file 10: Table S5, sheet genomes metagenomic analysis) with Bowtie aligner (same parameters, and the --best --strata option). A first analysis was done to explain the unmapped reads. The distribution of the mean quality of the reads for the cheese sample was evaluated for the mapped reads (see Additional file 16: Figure S6). The mean quality of the mapped reads was higher than 20 for more than 95% of the reads. Considering this, reads with a mean quality under the value of 20 were considered as “bad quality reads” and not considered for the metagenomic analyses. Unmapped good quality reads were then mapped on *Bos taurus* genomes with Bowtie (same parameters). Finally, an analysis of the unmapped good quality read percentage was performed using the same SOLiD technology (SOLiD v4) and the same read mapper (Bowtie) than the cheese analysis with a set of five bacterial genome re-sequencing projects (see Additional file 15: Table S9). While in this experiment 100% of the good quality reads should have mapped with their reference genome, only 80% of the good quality reads did, possibly due to sequencing errors in the reads.

Finally, genomes present in samples were identified from the second mapping results. Genomic regions that were less informative and/or that could have been acquired by gene transfer (intergenic regions, tRNA, rRNA, genes annotated as “transposase”, “integrase”, “IS”, “phage/prophage” or “plasmids”) were removed. We then quantified the percentage of the genome covered by at least one read, and the percentage of the genes covered by at least one read. A first filter was done by removing genomes to which less than 700 reads mapped and genomes with less than 20% of coding DNA sequences covered by at least one read. In order to estimate if the reads were evenly mapped across the genome, we computed the average number of reads per coding DNA sequences. (number of reads / number of coding DNA sequences). If the average number was > =10 and the observed percentage of coding DNA sequences covered by reads > 80% or the average number between 2 and 10 and the observed percentage of coding DNA sequences covered by reads > 70%, we considered the species to be present in the cheese. Finally, as reads were allowed to map several genomes, some reads could map to several reference genomes of the same species even when only one strain of this species is present in the sample. Therefore, to get more stringent results, only one reference genome was kept for each species or subspecies (the genome with the highest percentage of coding DNA sequences covered by reads). The relative abundances of the different genomes were calculated by dividing the number of reads assigned to each genome by the number of good quality reads. The raw SOLiD and Illumina read data for all samples has been deposited in the European Bioinformatics Institute (EBI) European Nucleotide Archive (ENA) under the accession number PRJEB6314.

## Electronic supplementary material

Additional file 1: Table S1: Origin and taxonomy of the bacterial isolates. (XLSX 96 KB)

Additional file 2: **Supplementary information document.** This supplementary information document details the genome assembly steps, from sequencing to the quality evaluation. (PDF 296 KB)

Additional file 3: Table S2: Establishment of the sequencing pools. (XLS 62 KB)

Additional file 4: Table S3: Evaluation of the genome clustering procedure and the quality of the genome drafts. (XLS 104 KB)

Additional file 5: Table S4: Bacterial species or subspecies with technological beneficial use in food products listed by Bourdichon and coworkers (2012). (XLSX 24 KB)

Additional file 6: Figure S1: Global phylogeny of 179 *Firmicutes* bacterial isolates, including 42 genomes from our project. (PNG 711 KB)

Additional file 7: Figure S2: Global phylogeny of 14 *Bacteroidetes* bacterial isolates, including 6 genomes from our project. (PNG 61 KB)

Additional file 8: Figure S3: Global phylogeny of 180 *Proteobacteria* bacterial isolates, including 50 genomes from our project. (PNG 1 MB)

Additional file 9: Figure S4: Global phylogeny of 84 *Actinobacteria* bacterial isolates, including 32 genomes from our project. (PNG 1 MB)

Additional file 10: Table S5: List of the genomes used for phylogenetic and metagenomic analyses. (XLSX 718 KB)

Additional file 11: Table S6: Proteins involved in the catabolism of D-galactonate in *Arthrobacter* strains. (DOCX 21 KB)

Additional file 12: Table S7: Genomic comparison of four *Streptococcus infantarius* subsp. *infantarius* strains. (DOCX 15 KB)

Additional file 13: Figure S5: Phylogeny of 14 LacZ proteins from *Streptococcus* strains. (PDF 58 KB)

Additional file 14: Table S8: Mapping of the sequencing reads from the metagenomic analysis of the cheese samples. (XLSX 27 KB)

Additional file 15: Table S9: Mapping of the sequencing reads from genomic analysis of already sequenced genome to assess the average level of reads that could not be mapped inherent to SOLiD technology. (XLSX 13 KB)

Additional file 16: Figure S6: Mean quality distribution of the reads from the metagenomic analysis of the cheese samples. (PDF 15 KB)
