# Supplementary material for: Construction of a dairy microbial genome catalog opens new perspectives for the metagenomic analysis of dairy fermented products
Source: BMC Genomics. 2014 Dec 13;15(1):1101. doi: 10.1186/1471-2164-15-1101 (PMC4320590; doi:10.1186/1471-2164-15-1101)
Supplement: Supplementary file 2 — Additional file 2: Supplementary information document. This supplementary information document details the genome assembly steps, from sequencing to the quality evaluation. (PDF 296 KB) [file 12864_2014_6903_MOESM2_ESM.pdf]

Genome assembly supplementary information

The 147 bacteria DNA samples were distributed in five independent sequencing pools containing each about 30 genomes. They were then sequenced by Illumina technology and assembled into contigs and scaffolds. To assign the contigs to their original genome, a co-variance clustering methods deriving from the method described by Le Chatelier et al. (2013)[1] was used. The procedure is described in the three next paragraphs.

Construction of six DNA SOLiD pools for clustering

Six DNA pools were created by mixing the 147 genomic DNA in a controlled way (about 90 strains in each pool, see Table S2). The presence or absence of each strain in the samples was represented by a vector ("signature vector"). The 1 digit corresponds to the presence of the strain in the SOLiD sequencing sample, and 0 to its absence, as illustrated below.

Genome signature vector

Genome Name	Illumina Sample ID	Sample SOLiD A	Sample SOLiD B	Sample SOLiD C	Sample SOLiD D	Sample SOLiD E	Sample SOLiD F
Exiguobacterium sibiricum NL25	3	0	1	0	1	1	1

In this example, the Strain NL25 from the Illumina pool 3 was added in the SOLiD sequencing samples B, D, E and F.

For each strain present in a specific Illumina pool, there is a unique signature vector. In addition, for improving the co-variance clustering procedure, we used the same signal vector for different strains belonging to the same genus, as illustrated bellow:

Strain	Illumina Pool	SOLiD Pool A for clustering	SOLiD Pool B for clustering	SOLiD Pool C for clustering	SOLiD Pool D for clustering	SOLiD Pool E for clustering	SOLiD Pool F for clustering
Arthrobacter arilaitensis 3M03	4	0	1	0	0	1	1
Arthrobacter arilaitensis GMPA29	5	0	1	0	0	1	1
Arthrobacter bergerei Ca106	2	0	1	0	0	1	1
Streptococcus infantarius subsp. infantarius 11FA	3	1	1	0	1	1	0
Streptococcus infantarius subsp. infantarius 3AG	5	1	1	0	1	1	0
Streptococcus macedonicus 679	2	1	1	0	1	1	0
Streptococcus salivarius CJ181	4	1	1	0	1	1	0

In this example, the strains belonging to the same genus (here *Arthrobacter* or *Streptococcus*) had the same signature vector. This was done in order to reduce the impact of conserved regions shared among strains from the same genus. As each Illumina pool contains only one strain from each genus and is treated independently, there will be no confusion during the clustering process.

Quantification of contigs coverage

The six SOLiD sequencing samples A, B, C, D, E and F, described in the previous paragraph were sequenced with single reads of 50 nucleotide length. In order to attribute Illumina contigs, previously assembled, to their original genome, the SOLiD reads were mapped on the Illumina contigs using BOWTIE mapper version 0.12.7[2] (color space mode, mapping size: 45 b, maximum mismatch tolerated: 1, maximum match limit: 10000). The contigs were then split into fragments of 1000 nucleotides and the fragments smaller than 100 nucleotides were discarded. The coverage value of each fragment was then calculated. It corresponds to the number of positions covered by at least one read divided by the size of the fragment as illustrated bellow:

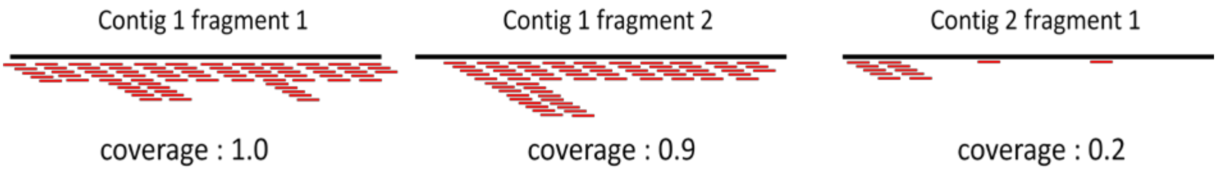

By this way, the coverage values range between 0.0 (absence) and 1.0 (presence). Finally a coverage matrix was created for each Illumina pool and used for the clustering process.

Clustering procedure

In order to re-assign the fragments to the corresponding genome, we compared the contig coverage vectors from the coverage matrix to the genome signature vectors using Pearson correlation coefficient. Only the Pearson correlation coefficients higher than 0.95 were considered. A contig was affected to a particular genome using the dominant assignation among the contig fragments. The assignation was discarded when no dominant assignation existed in the fragments of a scaffold. The clustering process is illustrated below:

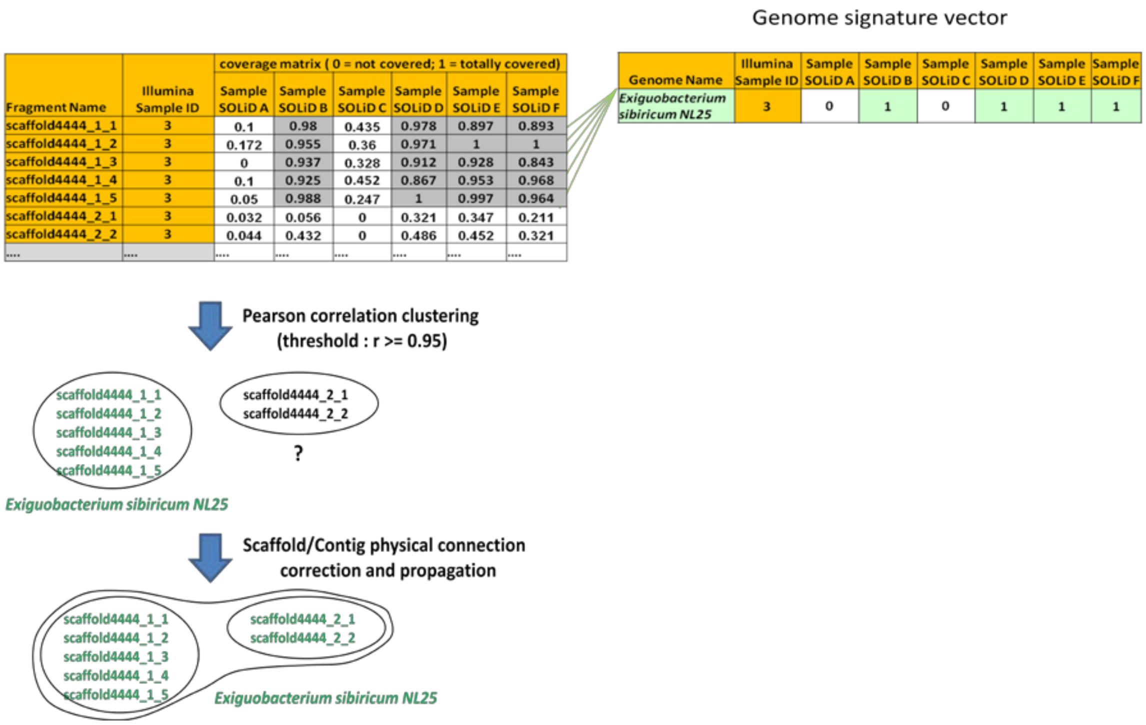

In this example, the scaffold 4444, from the Illumina sample 3, was divided in seven fragments (belonging to two contigs). Five of the fragments were assigned by clustering to the genome of the NL25 strain and the two others were not assigned at this level. This may be explained for example by a lower sequencing efficiency in this region. The physical connection of the fragments in the same scaffold was then used to recover them.

Evaluation of sequencing and clustering quality

Due to the high fragmentation level of the contigs of *Brochotrix thermosphacta* cH814 and *Citrobacter freundii* Pi15 in Illumina pool 1, their genomes were sequenced a second time in another Illumina pool. These replicates were used to evaluate the quality of the clustering process. This is illustrated in the following table, which shows the percentage of overlapping (in comparison to the largest genome):

	Cumulated contig size of the largest cluster (Mpb)	Cumulated contig size of the smallest cluster (Mpb)	Overlapping percentage of the clusters (smallest onto the largest)	Percentage of identity between the shared nucleotidic sequences
Brochotrix thermosphacta cH814	2.44	1.92	96.69	99.99
Citrobacter freundii Pi15	5.47	5.32	97.85	99.99

There was a high percentage of identity between the shared nucleotidic sequences (>99.99%), showing a high fidelity of the sequencing. The high percentage of overlapping of the clusters indicates that there was a good reassignment of the contigs to their genomes by the clustering method.

Chimeric evaluation criteria

To evaluate the potential contamination of our draft genomes with other genomes, we defined two supplementary criteria: the tetra-nucleotide frequency composition and the marker redundancy. These two criteria are explained below.

Tetra-nucleotide frequency composition

Numerous studies showed that tetra-nucleotide frequency in the contigs and scaffolds of a bacterial genome are conserved [3]. This signature is conserved at the species level and may be used to differentiate the contigs of strains belonging to different genera. Some clustering procedures use this principle to re-attribute the contigs of a metagenome to their original genome. With the MetaCluster software, a good specificity is achieved for contigs larger than 1000 nucleotides [4]. We used the same pipeline than in the MetaCluster software to calculate the tetra-nucleotide frequency vector for each contig and calculated the average Spearman correlation coefficient between the different tetra-nucleotide vectors of a draft genome. All the different tetra-nucleotides were counted for each contig of the draft genome. After that, the tetra-nucleotide values from each contig were divided by the size of the contig to obtain a tetra-nucleotide frequency vector for each contig. We finally computed the Spearman rho coefficient correlation between all the tetra-nucleotide vectors and calculated the average rho

Spearman coefficient value. An average Spearman rho value >= 0.6 was used to define a draft genome as homogenous in tetra-nucleotide composition. This threshold was relatively low, in order to take into account the tetra-nucleotide frequency biases due to short length contigs in highly fragmented genomes, and to the unspecific regions that have a different tetra-nucleotide signature in a genome (regions resulting from horizontal transfers, transposons, prophages).

Marker redundancy

Sorek et al. (2007) [5] proposed a list of 40 proteins present in all bacteria, which corresponding genes are not transferable and not duplicable within a genome. We used these proteins as phylogenetic markers, and created a high resolution tree, which was used to evaluate the possible contamination in each draft genome. As these markers should not be redundant, their duplication in a draft indicates a probable contamination

List of the 40 markers:

COG ID	Annotation	Fonction (NCBI)
COG0012	Predicted GTPase, probable translation factor	Translation, ribosomal structure and biogenesis
COG0016	Phenylalanine-tRNA synthetase alpha subunit	Translation, ribosomal structure and biogenesis
COG0018	Arginyl-tRNA synthetase	Translation, ribosomal structure and biogenesis
COG0048	Ribosomal protein S12	Translation, ribosomal structure and biogenesis
COG0049	Ribosomal protein S7	Translation, ribosomal structure and biogenesis
COG0052	Ribosomal protein S2	Translation, ribosomal structure and biogenesis
COG0080	Ribosomal protein L11	Translation, ribosomal structure and biogenesis
COG0081	Ribosomal protein L1	Translation, ribosomal structure and biogenesis
COG0085	DNA-directed RNA polymerase, beta subunit	Transcription
COG0088	Ribosomal protein L4	Translation, ribosomal structure and biogenesis
COG0087	Ribosomal protein L3	Translation, ribosomal structure and biogenesis
COG0090	Ribosomal protein L2	Translation, ribosomal structure and biogenesis
COG0091	Ribosomal proteinL22	Translation, ribosomal structure and biogenesis
COG0092	Ribosomal protein S3	Translation, ribosomal structure and biogenesis
COG0093	Ribosomal protein L14	Translation, ribosomal structure and biogenesis
COG0094	Ribosomal protein L5	Translation, ribosomal structure and biogenesis
COG0096	Ribosomal protein S8	Translation, ribosomal structure and biogenesis
COG0097	Ribosomal protein L6P/L9E	Translation, ribosomal structure and biogenesis
COG0098	Ribosomal protein S5	Translation, ribosomal structure and biogenesis
COG0099	Ribosomal protein S13	Translation, ribosomal structure and biogenesis
COG0100	Ribosomal protein S11	Translation, ribosomal structure and biogenesis
COG0102	Ribosomal protein L13	Translation, ribosomal structure and biogenesis

COG0103	Ribosomal protein S9	Translation, ribosomal structure and biogenesis
COG0124	Histidyl-tRNA synthetae	Translation, ribosomal structure and biogenesis
COG0172	Seryl-tRNA synthetase	Translation, ribosomal structure and biogenesis
COG0184	Ribosomal protein S15P/S13E	Translation, ribosomal structure and biogenesis
COG0185	Ribosomal protein S19	Translation, ribosomal structure and biogenesis
COG0186	Ribosomal protein S17	Translation, ribosomal structure and biogenesis
COG0197	Ribosomal protein SL16/L10E	Translation, ribosomal structure and biogenesis
COG0200	Ribosomal protein L15	Translation, ribosomal structure and biogenesis
COG0201	Preprotein translocase subunit SecY	Intracellular trafficking, secretion and vesicular transport
COG0202	DNA-directed RNA polymerase, alpha subunit	Transcription
COG0215	Cysteinyl-tRNA synthetase	Translation, ribosomal structure and biogenesis
COG0256	Ribosomal protein L18	Translation, ribosomal structure and biogenesis
COG0495	Leucyl-tRNA synthetase	Translation, ribosomal structure and biogenesis
COG0522	Ribosomal protein S4 and related proteins	Translation, ribosomal structure and biogenesis
COG0525	Valyl-tRNA synthetase	Translation, ribosomal structure and biogenesis
COG0533	Metal-dependent proteases with chaperone activity	Post-translational modification, protein turnover, chaperones
COG0541	Signal recognition particle GTPase	Intracellular trafficking, secretion and vesicular transport
COG0552	Signal recognition particle GTPase	Intracellular trafficking, secretion and vesicular transport

References

1. Le Chatelier E, Nielsen T, Qin J, Prifti E, Hildebrand F, Falony G, Almeida M, Arumugam M, Batto J-M, Kennedy S, Leonard P, Li J, Burgdorf K, Grarup N, Jørgensen T, Brandslund I, Nielsen HB, Juncker AS, Bertalan M, Levenez F, Pons N, Rasmussen S, Sunagawa S, Tap J, Tims S, Zoetendal EG, Brunak S, Clément K, Doré J, Kleerebezem M, et al.: **Richness of human gut microbiome correlates with metabolic markers.** *Nature* 2013, **500**:541–6.

2. Langmead B, Trapnell C, Pop M, Salzberg SL: **Ultrafast and memory-efficient alignment of short DNA sequences to the human genome.** *Genome biology* 2009, **10**:R25.

3. Karlin S, Ladunga I: **Comparisons of eukaryotic genomic sequences.** *Proceedings of the National Academy of Sciences of the United States of America* 1994, **91**:12832–6.

4. Yang B, Peng Y, Leung HC-M, Yiu S-M, Chen J-C, Chin FY-L: **Unsupervised binning of environmental genomic fragments based on an error robust selection of l-mers.** *BMC bioinformatics* 2010, **11 Suppl 2**(Suppl 2):S5.

5. Sorek R, Zhu Y, Creevey CJ, Francino MP, Bork P, Rubin EM: **Genome-wide experimental determination of barriers to horizontal gene transfer.** *Science (New York, NY)* 2007, **318**:1449–52.

6. Altschul S, Gish W, Miller W: **Basic local alignment search tool**. *Journal of molecular …* 1990, **215**:403–410.

7. Lindner MS, Kollock M, Zickmann F, Renard BY: **Analyzing genome coverage profiles with applications to quality control in metagenomics.** *Bioinformatics (Oxford, England)* 2013, **29**:1260–7.

8. Benjamini Y, Speed TP: **Summarizing and correcting the GC content bias in high-throughput sequencing.** *Nucleic acids research* 2012, **40**:e72.
