# Supplementary figures and images for: Construction of a dairy microbial genome catalog opens new perspectives for the metagenomic analysis of dairy fermented products
Source: BMC Genomics. 2014 Dec 13;15(1):1101. doi: 10.1186/1471-2164-15-1101 (PMC4320590; doi:10.1186/1471-2164-15-1101)

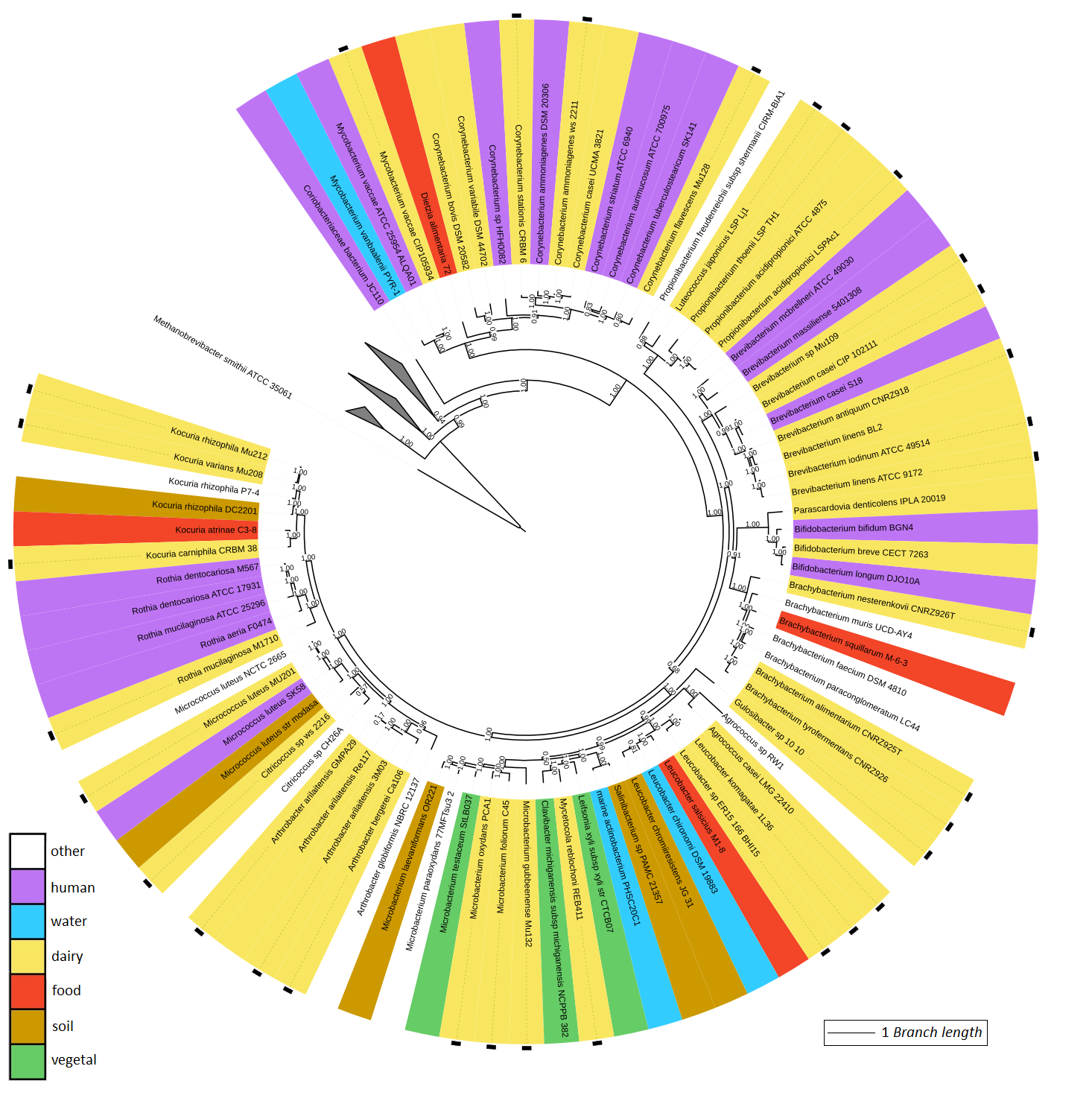

Supplement: Supplementary file 6 — Additional file 6: Figure S1: Global phylogeny of 179 Firmicutes bacterial isolates, including 42 genomes from our project. (PNG 711 KB) [file 12864_2014_6903_MOESM6_ESM.png]

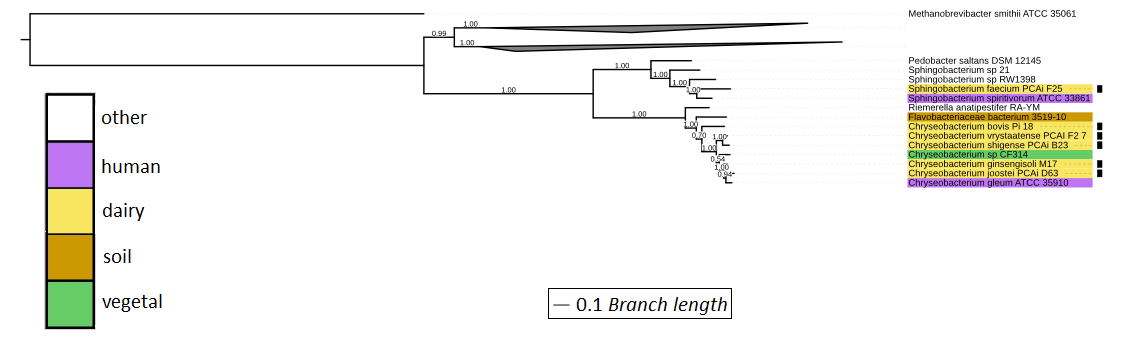

Supplement: Supplementary file 7 — Additional file 7: Figure S2: Global phylogeny of 14 Bacteroidetes bacterial isolates, including 6 genomes from our project. (PNG 61 KB) [file 12864_2014_6903_MOESM7_ESM.png]

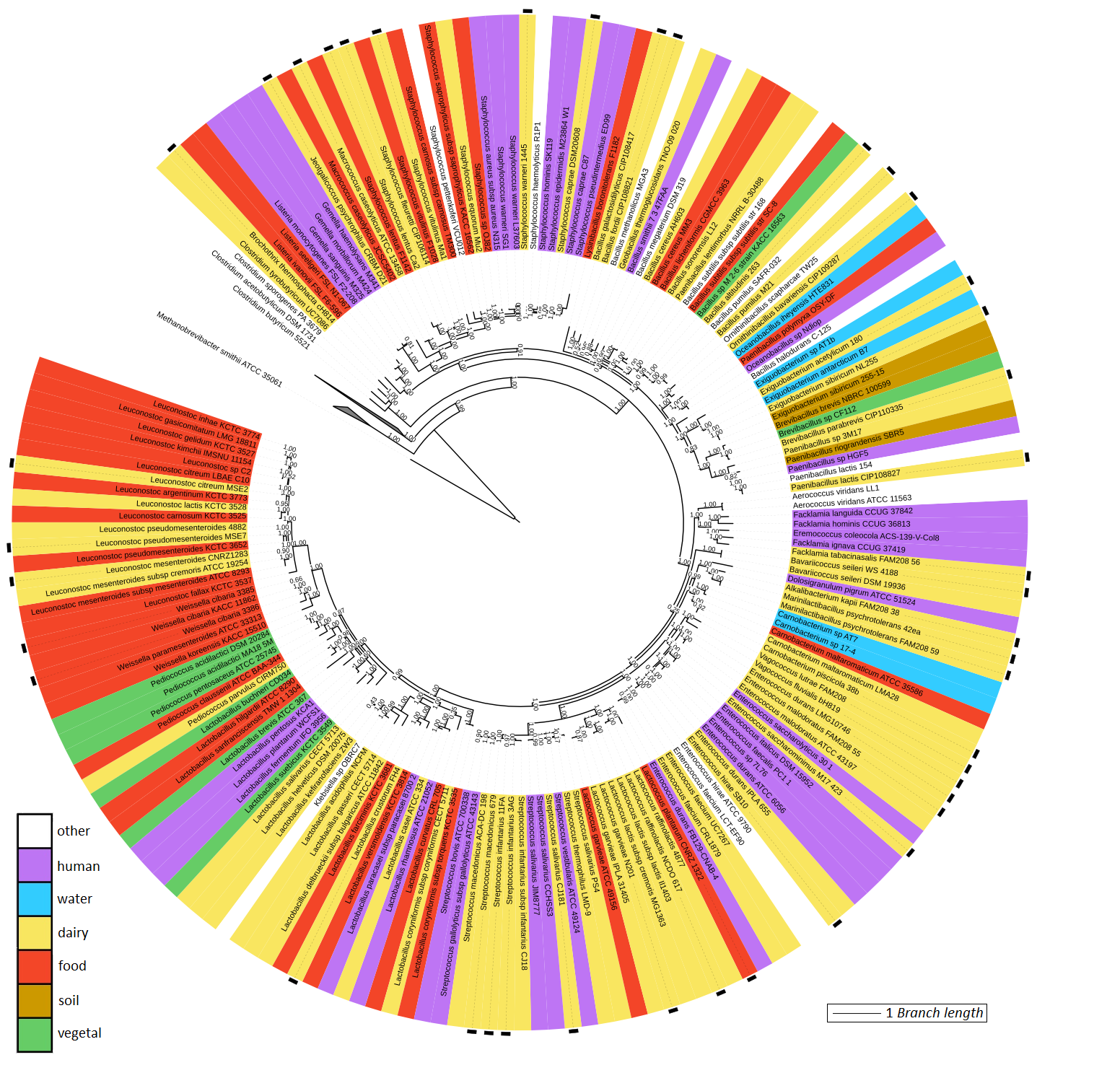

Supplement: Supplementary file 8 — Additional file 8: Figure S3: Global phylogeny of 180 Proteobacteria bacterial isolates, including 50 genomes from our project. (PNG 1 MB) [file 12864_2014_6903_MOESM8_ESM.png]

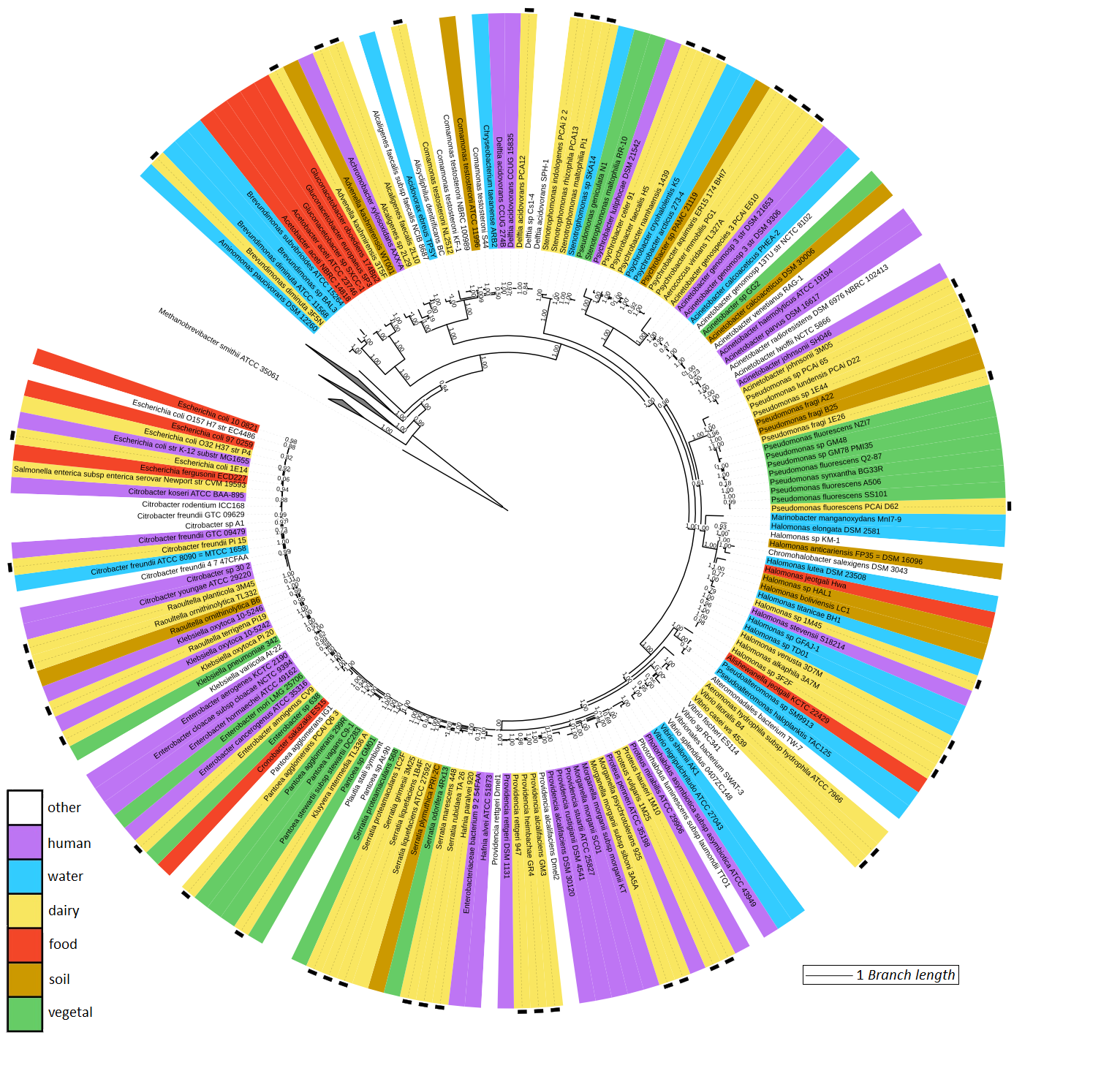

Supplement: Supplementary file 9 — Additional file 9: Figure S4: Global phylogeny of 84 Actinobacteria bacterial isolates, including 32 genomes from our project. (PNG 1 MB) [file 12864_2014_6903_MOESM9_ESM.png]

Fig. S6

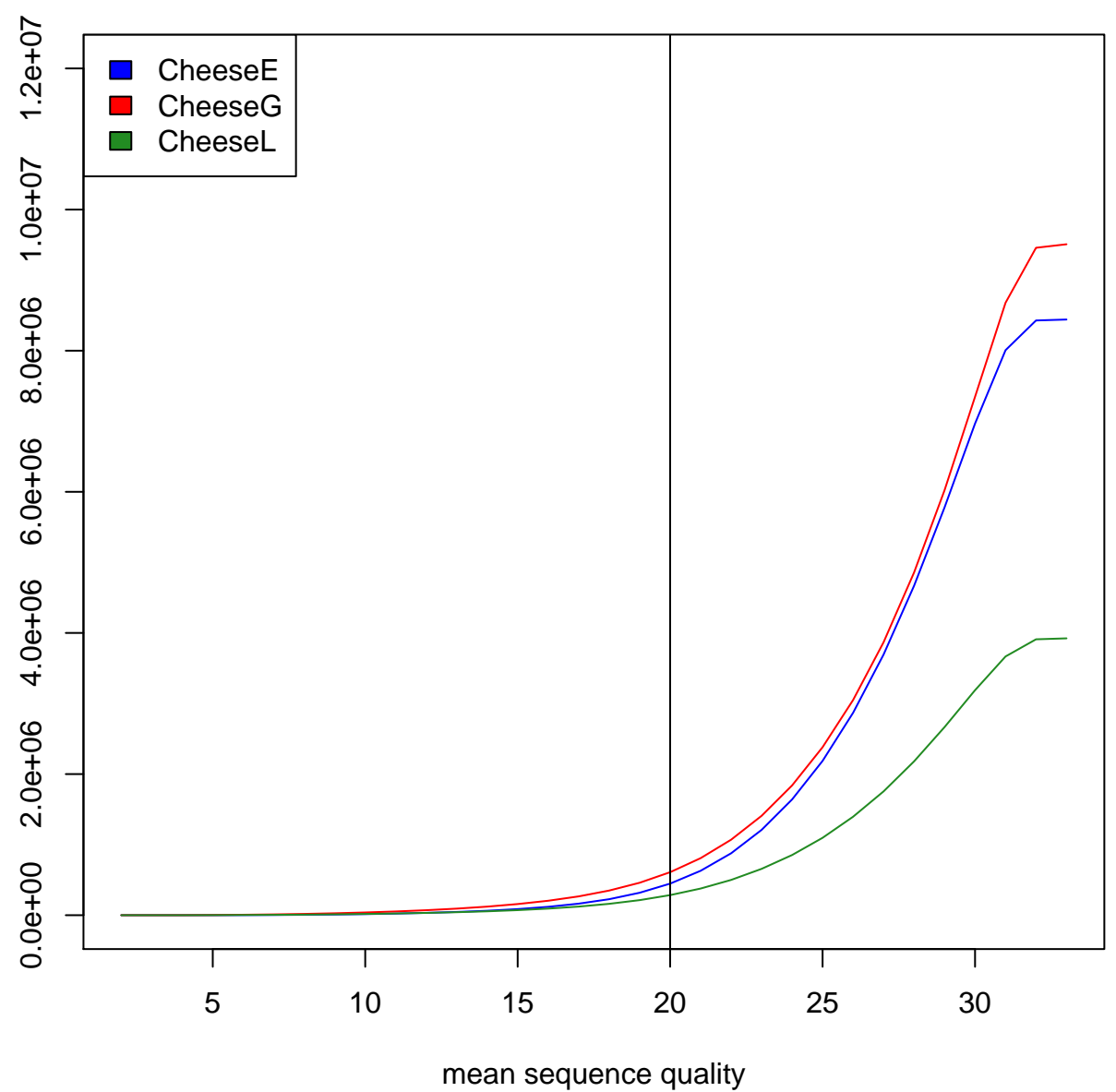

Supplement: Supplementary file 16 — Additional file 16: Figure S6: Mean quality distribution of the reads from the metagenomic analysis of the cheese samples. (PDF 15 KB) [file 12864_2014_6903_MOESM16_ESM.pdf]
