# Supplementary material for: Construction of a dairy microbial genome catalog opens new perspectives for the metagenomic analysis of dairy fermented products
Source: BMC Genomics. 2014 Dec 13;15(1):1101. doi: 10.1186/1471-2164-15-1101 (PMC4320590; doi:10.1186/1471-2164-15-1101)
Supplement: Supplementary file 11 — Additional file 11: Table S6: Proteins involved in the catabolism of D-galactonate in Arthrobacter strains. (DOCX 21 KB) [file 12864_2014_6903_MOESM11_ESM.docx]

Table S6. Proteins involved in the catabolism of D-galactonate in *Arthrobacter* strains

| Strain | Origin | Genbank project n° | Proteins involved in the catabolism of D-galactonate^1^ | | | | | Comment |
| --- | --- | --- | --- | --- | --- | --- | --- | --- |
|  |  |  | D-galactonate regulator, IclR family | 2-dehydro-3-deoxygalactonokinase  (EC 2.7.1.58) | 2-dehydro-3-deoxy-6-phosphogalactonate aldolase (EC 4.1.2.21) | galactonate dehydratase (E.C.4.2.1.6) | D-galactonate importer |  |
|  |  |  |  |  |  |  |  |  |
| *Arthrobacter arilaitensis* Re117 | cheese | PRJEB351 (present study) | **D-galactonate regulator, IclR family (100%)** | **2-dehydro-3-deoxygalactonokinase (100%)** | **2-dehydro-3-deoxy-6-phosphogalactonate aldolase (100%)** | **galactonate dehydratase (100%)** | **D-galactonate importer (100%)** | correspond to a cluster of five genes |
| *Arthrobacter* GMPA29 | cheese | PRJEB354 (present study) | D-galactonate regulator, IclR family (99%) | 2-dehydro-3-deoxygalactonokinase (98%) | 2-dehydro-3-deoxy-6-phosphogalactonate aldolase (98%) | galactonate dehydratase (100%) | D-galactonate importer (96%) | gene synteny identical to that of *Arthrobacter arilaitensis* Re117 |
| *Arthrobacter bergerei* Ca106 | cheese | PRJEB277 (present study) | D-galactonate regulator, IclR family (90%) | 2-dehydro-3-deoxygalactonokinase (65%) | 2-dehydro-3-deoxy-6-phosphogalactonate aldolase (80%) | galactonate dehydratase (97%) | D-galactonate importer (90%) | gene synteny identical to that of *Arthrobacter arilaitensis* Re117 |
| *Arthrobacter arilaitensis* 3M03 | cheese | PRJEB261 (present study) | D-galactonate regulator, IclR family (100%) | 2-dehydro-3-deoxygalactonokinase (100%) | 2-dehydro-3-deoxy-6-phosphogalactonate aldolase (100%) | galactonate dehydratase (99%) | D-galactonate importer (96%) | gene synteny identical to that *of Arthrobacter arilaitensis* Re117 |
| *Arthrobacter sp.* FB24 | soil contaminated with heavy metals and aromatic solvents | PRJNA58141 | / | / | / | / | / | / |
| *Arthrobacter sp.* Rue61a | sludge of a biological wastewater treatment plant | PRJNA174511 | / | / | / | / | / | / |
| *Arthrobacter sp*. 131MFCol6.1 | rhizosphere / endosphere of Arabidopsis thaliana | PRJNA199313 | / | / | hypothetical protein WP_018775435  (40%) | galactonate dehydratase WP_018775436  (50%) | / | / |
| *Arthrobacter sp.* 135MFCol5.1 | rhizosphere / endosphere of Arabidopsis thaliana | PRJNA199310 | / | / | / | / | / | / |
| *Arthrobacter sp.* 161MFSha2.1 | rhizosphere / endosphere of Arabidopsis thaliana | PRJNA199314 | / | / | / | / | / | / |
| *Arthrobacter sp.* 162MFSha1.1 | rhizosphere / endosphere of Arabidopsis thaliana | PRJNA199312 | / | / | / | galactonate dehydratase WP_018770859  (50%) | / | / |
| *Arthrobacter sp.* M2012083 | tobacco waste | PRJNA198986 | / | / | / | / | / | / |
| *Arthrobacter sp.* SJCon | pesticide-contaminated site | PRJNA187581 | / | / | / | / | / | / |
| *Arthrobacter sp.* TB 23 | Antarctic sponge | PRJNA199581 | / | / | / | galactonate dehydratase WP_019481918  (52%) | / | / |
| *Arthrobacter crystallopoietes* BAB-32 | soil sample | PRJNA184765 | / | / | / | / | / | / |
| *Arthrobacter globiformis* NBRC 12137 | soil | PRJNA78817 | / | / | / | / | / | / |
| *Arthrobacter phenanthrenivorans* Sphe3 | creosote contaminated soil | PRJNA63629 | / | / | / | / | / | / |
| *Arthrobacter chlorophenolicus* A6 | soil | PRJNA58969 | / | / | / | / | / | / |
| *Arthrobacter aurescens* TC1 | spill site soil | PRJNA58109 | / | / | / | / | / | / |
| *Arthrobacter gangotriensis* Lz1y | penguin rookery soil sample collected in Antarctica | PRJNA195197 | / | / | / | / | / |  |

^1^Homologs of the *Arthrobacter arilaitensis* Re117 proteins involved in the catabolism of D-galactonate (indicated in bold) were searched using the BLASTP program (<http://blast.ncbi.nlm.nih.gov/>). The percentages in brackets indicate the corresponding identity levels. No other proteins involved in the catabolism of D-galactonate could be evidenced by examination of the annotation data of the *Arthrobacter* genomes
