# Supplementary material for: Construction of a dairy microbial genome catalog opens new perspectives for the metagenomic analysis of dairy fermented products
Source: BMC Genomics. 2014 Dec 13;15(1):1101. doi: 10.1186/1471-2164-15-1101 (PMC4320590; doi:10.1186/1471-2164-15-1101)
Supplement: Supplementary file 12 — Additional file 12: Table S7: Genomic comparison of four Streptococcus infantarius subsp. infantarius strains. (DOCX 15 KB) [file 12864_2014_6903_MOESM12_ESM.docx]

Table S7. Genomic comparison of four *Streptococcus infantarius* subsp. *infantarius* strains. Indicated are the numbers of common genes defined by double best hit analysis and, given in brackets, the corresponding mean percentages of identity at the nucleotide level.

|  | ATCC BAA-102 (infant feces) | CJ18 (eastern African fermented milk) | 3AG (western African fermented milk) | 11FA (western African fermented milk) |
| --- | --- | --- | --- | --- |
| ATCC BAA-102 (infant feces) | 1936 (100%) | 1726 (99.28%) | 1636 (98.84%) | 1613 (98.87%) |
| CJ18 (eastern African fermented milk) |  | 2009 (100%) | 1640 (98.87%) | 1625 (98.89%) |
| 3AG (western African fermented milk) |  |  | 1864 (100%) | 1768 (99.69%) |
| 11FA (western African fermented milk) |  |  |  | 1893 (100%) |
