# Supplementary material for: Construction of a dairy microbial genome catalog opens new perspectives for the metagenomic analysis of dairy fermented products
Source: BMC Genomics. 2014 Dec 13;15(1):1101. doi: 10.1186/1471-2164-15-1101 (PMC4320590; doi:10.1186/1471-2164-15-1101)

Evidence for an independent horizontal *lacZ* gene transfer in western and eastern African *S. intantarius* subsp. *infantarius* strains. (A) Phylogenic tree obtained with LacZ present in African *S. infantarius* subsp. *infantarius* (Sii) and in streptococcal species of the salivarius group (*S. thermophilus*, Sth; *S. salivarius*, Ssa; *S. vestibularis*, Sve; *S. sp.*, Ssp). (B) Phylogenic tree obtained from distance matrices based on the nucleotide identity of the core parts in by pair genome comparisons of the four *S. infantarius* subsp. *infantarius* strains

Genome sequences considered:

>Sii_3AG	this work
>Sii_CJ18	AEZ62269.1
>Ssa_57.I	AEJ53119.1
>Ssa_CCHSS3	CCB93019.1
>Ssa_JIM8777	CCB95710.1
>Ssa_M18	EGX29678.1
>Ssa_PS4]	EIC81642.1
>Ssp_C150	EFX55369.1
>Sth_CJ181	this work
>Sth_JIM8232*	CCC20291.1
>Sth_LMG18311*	AAV61011.1
>Sve_ATCC49124	EFX96471.1
>Sve_F0396	EFQ59374.1

* sequences 100% identical to those of other *S. thermophilus* strains: CNRZ1066 (AAV62934.1) , LMD-9 (ABJ66539.1), ND03 (ADQ63372.1), MN-ZLW-002 (AFJ83754.1), CAG:236 (CDA38473.1) and TH1435 (ETE40796.1)

Sequences were aligned by MUSCLE, curated by Gblocks, the phylogenic trees were built by PhyML and rendered by Tree Rendering which are connected in Phylogeny.fr (Dereeper A., Guignon V., Blanc G., Audic S., Buffet S., Chevenet F., Dufayard J.F., Guindon S., Lefort V., Lescot M., Claverie J.M., Gascuel O. *Phylogeny.fr: robust phylogenetic analysis for the non-specialist.* Nucleic Acids Res. 2008 Jul 1;36

A

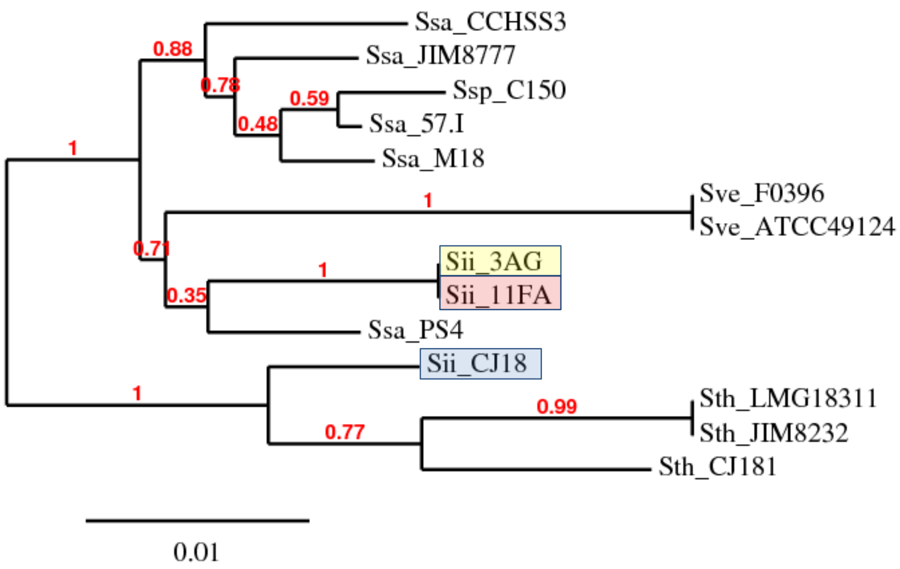

B

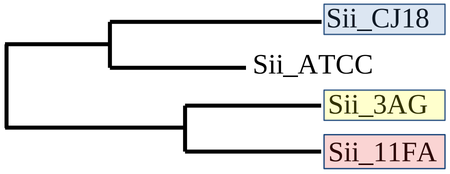

Supplement: Supplementary file 13 — Additional file 13: Figure S5: Phylogeny of 14 LacZ proteins from Streptococcus strains. (PDF 58 KB) [file 12864_2014_6903_MOESM13_ESM.pdf]
